# Application of deep learning models integrating attention mechanisms in operating room nursing quality and postoperative risk assessment for tumors

**DOI:** 10.3389/fonc.2026.1834175

**Published:** 2026-07-29

**Authors:** Zhiping Liu, Mingming Sun, Zhendan Xu

**Affiliations:** 1 School of Mathematics and Statistics, Shanxi Datong University, Datong, Shanxi, China; 2 Xinxiang Central Hospital, Department of Anesthesiology and Perioperative Medicine, The Fourth Clinical College of Xinxiang Medical College, Xinxiang, China; 3 Xinxiang Central Hospital, The Fourth Clinical College of Xinxiang Medical University, Xinxiang, China

**Keywords:** attention mechanisms, deep learning, operating room nursing, postoperative risk assessment, tumor surgery

## Abstract

**Introduction:**

This study proposes the Counterfactual Risk Assessor, a deep learning framework integrating attention mechanisms for operating room nursing quality assessment and postoperative risk prediction in tumor surgery patients. The framework is motivated by the need to model heterogeneous perioperative information and dynamic clinical events more effectively than static risk assessment approaches.

**Methods:**

It contains three main modules: Low Dimensional Manifold Projection, Event driven Segmentation Router, and Probabilistic Outcome Modeler. The projection module learns compact representations from high dimensional perioperative data while preserving clinically relevant structure. The segmentation router identifies event associated time windows and emphasizes informative signals such as abnormal vital signs, nursing interventions, medication administration, transfusion, and postoperative observations. The outcome modeler then estimates future risk probabilities using attention based representations and uncertainty aware prediction. Counterfactual Pacing is used to examine plausible alternative perioperative scenarios, and uncertainty propagation is applied to quantify prediction confidence.

**Results and Discussion:**

Experimental results show that the proposed framework achieves better accuracy, F1 score, AUROC, and AUPRC than clinical, statistical, machine learning, and deep learning baselines across the evaluated datasets. These findings suggest that event aware representation learning may provide useful support for postoperative risk stratification and nursing quality evaluation. The proposed framework should be regarded as a decision support method rather than a replacement for clinical judgment, and further prospective validation, external multicenter testing, and workflow integration are required before routine clinical application.

## 1 Introduction

Operating room nursing quality assessment and postoperative risk prediction are important components of perioperative management for tumor surgery patients. Tumor surgeries often involve complex procedures, heterogeneous patient characteristics, variable intraoperative conditions, and rapidly changing postoperative states. These factors make it difficult to evaluate nursing quality and estimate postoperative risk using only static or manually defined indicators (1). Accurate risk estimation may help clinicians identify patients requiring closer monitoring, while structured nursing quality assessment may support quality improvement in operating room care (2). In this context, computational models that can integrate perioperative information from multiple sources have attracted increasing attention (3). Attention mechanisms are particularly relevant because they allow predictive models to assign different weights to clinically informative features, time windows, or event signals (4).

Early studies on nursing quality evaluation and postoperative risk assessment commonly relied on rule based systems. These methods used predefined criteria, clinical guidelines, or expert knowledge to support decision making (5). Although rule based approaches are interpretable and easy to implement, their performance is limited when patient status changes dynamically or when unexpected intraoperative events occur (6). Their dependence on fixed rules also restricts adaptability across different surgical contexts, patient populations, and institutional workflows (7).

Machine learning methods were later introduced to learn statistical patterns from clinical data. Decision trees, ensemble methods, and other conventional algorithms improved predictive flexibility compared with rule based systems (8). These approaches can model nonlinear associations among perioperative variables and have been used in different healthcare prediction tasks (9). However, they often depend on manual feature engineering, careful variable selection, and extensive domain expertise (10). In perioperative settings, this limitation is important because relevant information may be distributed across demographic variables, laboratory indicators, anesthesia records, intraoperative monitoring data, nursing interventions, and postoperative observations.

Deep learning models provide an alternative by learning hierarchical representations from complex healthcare data (11). When combined with attention mechanisms, these models can emphasize clinically relevant features and improve the transparency of risk prediction to some extent (12). Existing studies have shown the potential of attention based models for handling heterogeneous clinical information and sequential data (13). Nevertheless, several challenges remain for postoperative risk prediction and nursing quality assessment. First, perioperative data are often high dimensional, noisy, and partially redundant. Second, clinically important information is frequently concentrated around specific events, such as abnormal vital signs, blood loss, medication administration, transfusion, emergency nursing interventions, or early postoperative complications. Third, model outputs should reflect uncertainty because clinical records may be incomplete and patient responses may vary substantially (14, 15).

To address these issues, this study proposes the Counterfactual Risk Assessor, a deep learning framework integrating attention mechanisms for operating room nursing quality assessment and postoperative risk prediction in tumor surgery patients. The framework includes three main modules. The Low Dimensional Manifold Projection learns compact perioperative representations while preserving clinically meaningful structures. The Event driven Segmentation Router identifies event associated temporal segments and emphasizes informative clinical signals. The Probabilistic Outcome Modeler estimates nursing quality and postoperative risk using attention based representations with uncertainty information. Counterfactual pacing is further used to examine plausible alternative perioperative scenarios, and uncertainty propagation is incorporated to support confidence aware prediction.

The main contributions are summarized as follows:

- A unified framework is developed for nursing quality assessment and postoperative risk prediction by integrating low dimensional representation learning, event driven segmentation, attention mechanisms, and probabilistic outcome modeling.
- An event aware routing strategy is introduced to emphasize clinically meaningful perioperative signals and reduce the influence of noisy or redundant information.
- Comprehensive experiments are conducted using clinical, statistical, machine learning, and deep learning baselines, with evaluation metrics including accuracy, F1 score, AUROC, and AUPRC.

## 2 Related work

### 2.1 Deep learning in healthcare

The integration of deep learning models in healthcare has revolutionized the way medical data is analyzed and interpreted. Deep learning, a subset of machine learning, utilizes neural networks with multiple layers to model complex patterns in data (10). In the context of healthcare, these models have been employed to enhance diagnostic accuracy, predict patient outcomes, and optimize treatment plans (11). The application of deep learning in operating room nursing quality and postoperative risk assessment for tumors is particularly promising due to its ability to process vast amounts of data and identify subtle patterns that may not be apparent to human clinicians (12). One of the primary advantages of deep learning in healthcare is its capacity to handle diverse data types, including medical imaging, electronic health records (EHRs), and genomic data (13). Convolutional neural networks (CNNs) have been extensively used in medical imaging to detect tumors, classify cancer types, and assess tumor progression (14). These models can analyze images with high precision, often surpassing human performance in specific tasks (15). In the operating room, deep learning models can assist in real time monitoring of patient vitals and surgical procedures, ensuring high quality nursing care and timely interventions (16). Moreover, deep learning models can be trained to predict postoperative risks by analyzing preoperative and intraoperative data (17). Recurrent neural networks (RNNs) and long short term memory (LSTM) networks are particularly suited for this task due to their ability to process sequential data (18). By examining patient history, surgical details, and intraoperative events, these models can provide risk assessments that inform postoperative care plans, potentially reducing complications and improving patient outcomes (19). The integration of attention mechanisms in deep learning models further enhances their applicability in healthcare (20). Attention mechanisms allow models to focus on specific parts of the input data, improving interpretability and performance (21). In the context of operating room nursing and postoperative risk assessment, attention mechanisms can highlight critical features in patient data, such as abnormal vital signs or significant surgical events, enabling more accurate predictions and timely interventions (22). Despite the potential benefits, the implementation of deep learning in healthcare faces several challenges (23). Data privacy and security are paramount concerns, as medical data is highly sensitive (24). Ensuring the ethical use of patient data and compliance with regulations such as the Health Insurance Portability and Accountability Act (HIPAA) is crucial (25). The interpretability of deep learning models continues to pose a significant challenge, given that these models are frequently regarded as “black boxes” (26). Developing methods to elucidate model decisions is essential for gaining clinician trust and facilitating clinical adoption (27).

### 2.2 Attention mechanisms in deep learning

Attention mechanisms have emerged as a pivotal component in the advancement of deep learning models, particularly in the field of natural language processing (NLP) (28). These mechanisms enable models to dynamically focus on specific parts of the input data, thereby enhancing their ability to capture relevant information and improve performance (29). The application of attention mechanisms extends beyond NLP, finding utility in various domains, including healthcare, where they contribute to more accurate and interpretable models (30). In the context of operating room nursing quality and postoperative risk assessment for tumors, attention mechanisms play a crucial role in refining deep learning models (10). By allowing models to prioritize certain features or data segments, attention mechanisms enhance the interpretability of predictions, making them more actionable for healthcare professionals (11). In analyzing patient data, attention mechanisms can highlight critical variables such as abnormal lab results or significant changes in vital signs, which are essential for assessing postoperative risks (12). The self attention mechanism, a variant of attention, has gained prominence due to its ability to capture dependencies across different parts of the input data (13). This mechanism is particularly beneficial in healthcare applications where patient data is often sequential and interdependent (14). Self attention allows models to weigh the importance of each data point relative to others, facilitating a comprehensive understanding of patient conditions and potential risks (15). In the operating room, this capability can be leveraged to monitor patient status in real time, ensuring high quality nursing care and timely interventions (16). Furthermore, attention mechanisms contribute to the development of more robust and generalizable models (17). By focusing on relevant features, these mechanisms help mitigate the impact of noise and irrelevant data, which is common in medical datasets (18). This results in models that are not only more accurate but also more resilient to variations in data quality and distribution (19). In the context of postoperative risk assessment, attention enhanced models can provide reliable predictions even in the presence of incomplete or noisy data, thereby supporting informed decision making in clinical settings (20). The integration of attention mechanisms in deep learning models also facilitates the development of explainable AI systems (21). By providing insights into which features or data segments influenced a model’s prediction, attention mechanisms enhance the transparency of AI systems, fostering trust among healthcare professionals (22). This is particularly important in clinical applications where understanding the rationale behind a model’s decision is crucial for patient safety and care (23).

### 2.3 Postoperative risk assessment models

Postoperative risk assessment is a critical component of surgical care, aimed at identifying patients at risk of complications following surgery (24). Accurate risk assessment enables healthcare providers to implement targeted interventions, optimize resource allocation, and improve patient outcomes (25). The advent of deep learning models has significantly advanced the field of postoperative risk assessment, offering new opportunities for precision medicine and personalized care (Gillespie et al. (26). Deep learning models, with their ability to process large volumes of complex data, are well suited for postoperative risk assessment (Gutierres et al. (27). These models can analyze diverse data sources, including electronic health records (EHRs), medical imaging, and intraoperative data, to generate comprehensive risk profiles for individual patients (28). By leveraging the power of neural networks, deep learning models can identify intricate patterns and interactions within the data that may be indicative of postoperative complications (29). Incorporating attention mechanisms into deep learning models further enhances their utility in postoperative risk assessment (30). Attention mechanisms allow models to focus on the most relevant features of the data, improving the accuracy and interpretability of risk predictions (10). In assessing the risk of surgical site infections, attention mechanisms can highlight key factors such as patient comorbidities, surgical duration, and intraoperative blood loss, providing valuable insights for clinicians (11). The use of deep learning models in postoperative risk assessment also facilitates the development of dynamic risk prediction tools (12). Unlike traditional risk assessment models, which often rely on static risk factors, deep learning models can continuously update risk predictions based on new data (13). This dynamic capability is particularly beneficial in the postoperative period, where patient conditions can change rapidly (14). By providing real time risk assessments, deep learning models support proactive clinical decision making and timely interventions (15). Despite the potential benefits, the implementation of deep learning models for postoperative risk assessment faces several challenges (16). Ensuring the quality and completeness of input data is essential for accurate risk predictions (17). The interpretability of model outputs continues to be a significant concern, as clinicians must comprehend the factors influencing risk predictions to make informed decisions (18). Addressing these challenges requires ongoing collaboration between data scientists, clinicians, and healthcare organizations to develop robust, transparent, and clinically relevant models (19). The integration of deep learning models in postoperative risk assessment represents a significant advancement in surgical care (20). By providing accurate, dynamic, and interpretable risk predictions, these models have the potential to transform postoperative management, reduce complications, and enhance patient outcomes (21). As research in this area continues to evolve, the development of innovative deep learning models will play a crucial role in advancing precision medicine and improving the quality of surgical care (22).

## 3 Methods

### 3.1 Overview

This section presents the methodological framework for operating room nursing quality assessment and postoperative risk prediction in tumor surgery patients. The proposed framework, named the Counterfactual Risk Assessor, is designed to integrate heterogeneous perioperative information, capture event associated clinical patterns, and generate uncertainty aware risk estimates.

The Counterfactual Risk Assessor consists of three modules: the Low Dimensional Manifold Projection, the Event driven Segmentation Router, and the Probabilistic Outcome Modeler. The Low Dimensional Manifold Projection maps high dimensional perioperative variables into a compact latent representation while preserving clinically meaningful relationships among patient characteristics, surgical factors, nursing interventions, and postoperative observations. This step reduces redundant information and provides a stable representation for subsequent modeling. The Event driven Segmentation Router processes perioperative time series according to clinically relevant events and temporal context. Instead of treating all time points as equally informative, this module emphasizes event associated windows, such as abnormal vital signs, medication administration, transfusion, emergency nursing intervention, procedure changes, and early postoperative observations. The routed representation is then aggregated into an event aware patient level representation for risk prediction. The Probabilistic Outcome Modeler estimates nursing quality and postoperative risk based on the event aware representation. Attention mechanisms are used to assign different weights to clinically informative features or temporal components. The model also incorporates uncertainty estimation so that prediction confidence can be considered together with the predicted outcome probability. The framework includes counterfactual pacing and uncertainty propagation. Counterfactual pacing is used to examine plausible alternative perioperative scenarios under constrained perturbations of input variables. Uncertainty propagation quantifies the variability of model predictions caused by incomplete records, noisy measurements, and heterogeneous patient responses. These strategies are intended to support more cautious interpretation of model outputs rather than to replace clinical decision making.

The following sections provide the formal problem definition and mathematical preliminaries in Section 3.2, describe the Counterfactual Risk Assessor in Section 3.3, and explain counterfactual pacing and uncertainty propagation in Section 3.4. The proposed method aims to provide an event aware and uncertainty aware decision support framework for perioperative risk assessment and nursing quality evaluation.

### 3.2 Preliminaries

This section formalizes the problem of evaluating nursing quality and postoperative risk in tumor surgeries through deep learning models with attention mechanisms. The goal is to establish a framework that effectively models the complex interactions and dependencies within the operating room environment, integrating diverse data sources and employing advanced modeling techniques to predict outcomes and assess risks.

Let $D={\left\{\left(x_{i},y_{i}\right)\right\}}_{i=1}^{N}$ denote the dataset, where *x_i_* represents the input features for the *i*-th surgical case, and *y_i_* is the corresponding outcome or risk label. The input features *x_i_* are decomposed into components such as patient demographics, intraoperative metrics, and postoperative observations, expressed as $x_{i}=\left(x_{i}^{p},x_{i}^{o},x_{i}^{r}\right)$.

The task involves learning a function f:X→Y that maps the input space X to the output space Y, where Y comprises possible outcomes or risk levels. This function is parameterized by a deep learning model incorporating attention mechanisms to emphasize relevant features and interactions.

To capture the temporal dynamics and event driven nature of the operating room, a time indexed sequence of events ${\left\{e_{t}\right\}}_{t=1}^{T}$ is introduced, with each event *e_t_* characterized by features *ϕ*(*e_t_*). The Event driven Segmentation Router processes this sequence, segmenting it into meaningful intervals for analysis.

The Counterfactual Risk Assessor evaluates potential outcomes under hypothetical scenarios, using counterfactual reasoning to assess how variable changes could impact overall risk. Let $C\left(x_{i}\right)$ denote the counterfactual scenario derived from the original input *x_i_*. The model assesses risk under this scenario using a probabilistic framework.

The Low Dimensional Manifold Projection ensures learned representations adhere to constraints derived from domain knowledge and empirical observations. These constraints are formulated as inequalities $C_{m}\left(x_{i}\right)\leq 0$, where $C_{m}$ represents manifold constraints.

The Probabilistic Outcome Modeler estimates the likelihood of different outcomes given input features and attention modulated representations, modeling the conditional probability distribution $P(y_{i}|x_{i},\theta )$, where *θ* denotes model parameters.

To incorporate uncertainty into predictions, a Bayesian framework is utilized, allowing uncertainty propagation through the model. This involves estimating posterior distributions over model parameters, denoted as P(θ|D), and integrating these distributions into the risk assessment process.

This approach provides a comprehensive assessment of nursing quality and postoperative risk by leveraging deep learning models and attention mechanisms. The integration of counterfactual reasoning and uncertainty propagation enhances the model’s ability to offer actionable insights and support decision making in the operating room context.

### 3.3 Counterfactual risk assessor

**Figure 1:**
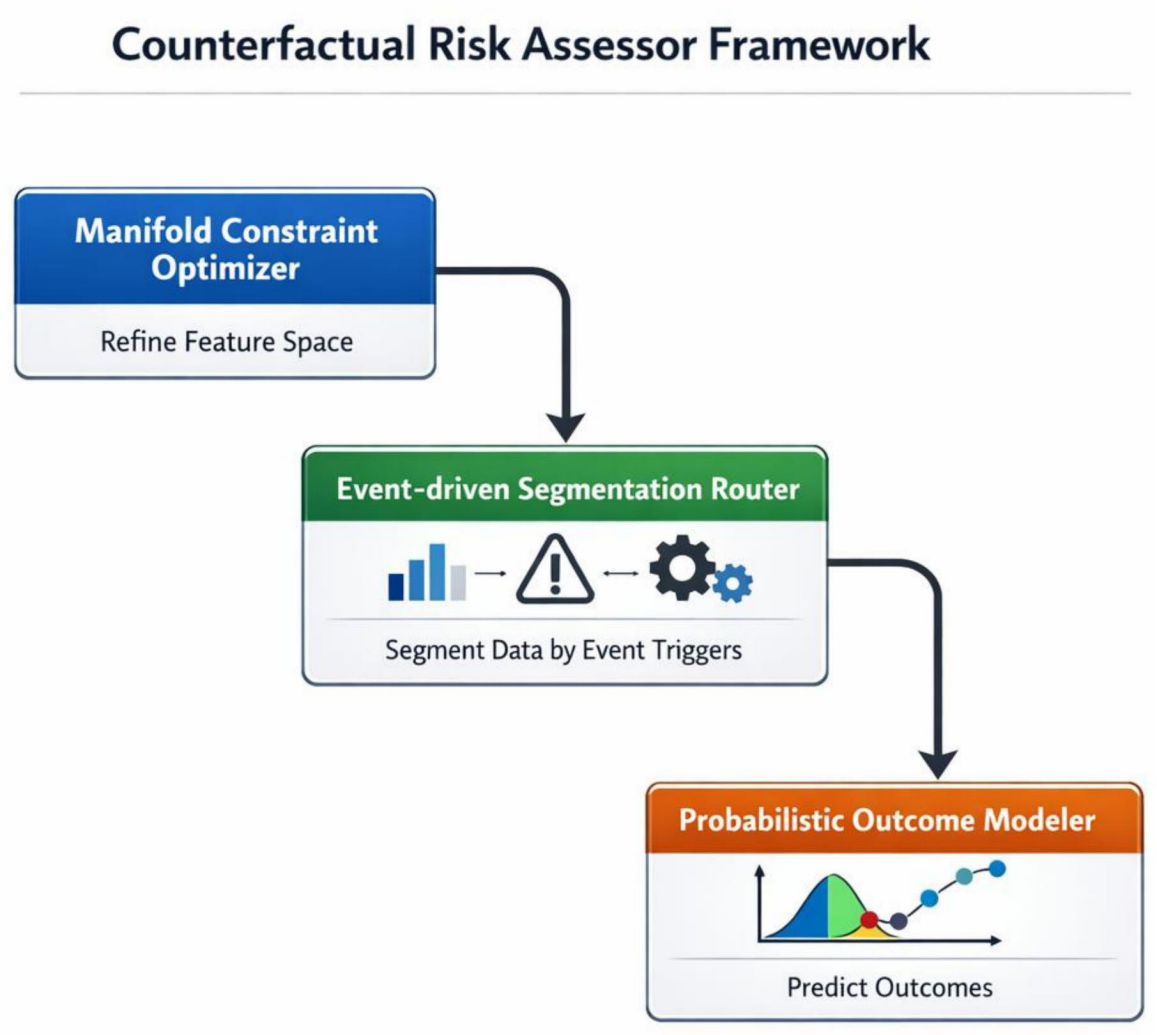
The counterfactual risk assessor framework integrates three modules to enhance operating room nursing quality and assess postoperative risk. The Low Dimensional Manifold Projection refines the feature space, improving model generalization. The Event driven Segmentation Router dynamically segments data based on real time events, while the Probabilistic Outcome Modeler predicts postoperative outcomes, incorporating uncertainties and patient characteristics.

As shown in **Figure 1**, In this section, we introduce the Counterfactual Risk Assessor, a novel model designed to enhance the quality of nursing in operating rooms and assess postoperative risks for tumor patients by integrating deep learning with attention mechanisms. The model is structured around three core modules: the Low Dimensional Manifold Projection, the Event driven Segmentation Router, and the Probabilistic Outcome Modeler. Each module plays a critical role in processing and analyzing data to provide accurate risk assessments and improve nursing interventions.

**Figure 2:**
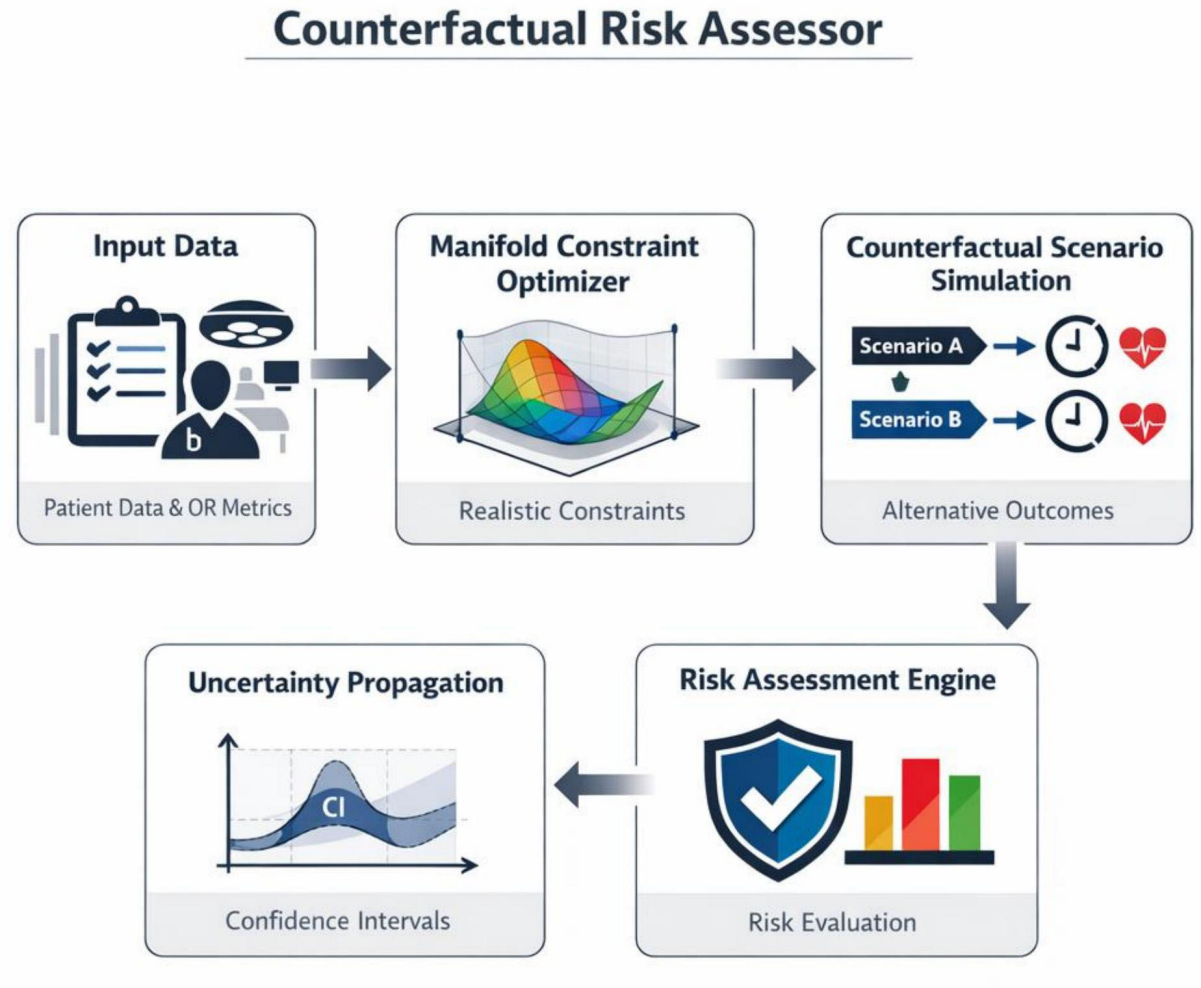
The diagram illustrates a deep learning framework for evaluating nursing quality and postoperative risk in tumor surgeries. Input features, including patient demographics, intraoperative metrics, and postoperative observations, are processed through feature extraction and attention mechanisms. The model outputs assessments of nursing quality and predictions of postoperative risk, leveraging complex interactions within the operating room environment.

Low Dimensional Manifold Projection: As shown in **Figure 2**, The Low Dimensional Manifold Projection is responsible for mapping high dimensional patient data into a lower dimensional manifold that preserves essential features while discarding noise. The Low Dimensional Manifold Projection was introduced because perioperative clinical data are typically high dimensional, heterogeneous, noisy, and partially redundant. In the operating room setting, a single surgical case may contain demographic variables, comorbidity indicators, laboratory measurements, intraoperative vital signs, medication records, nursing intervention records, and postoperative observations. Directly modeling all raw variables may increase computational burden, amplify noise, and lead to unstable risk estimation, especially when the number of clinically meaningful events is smaller than the number of recorded variables. Therefore, dimensionality reduction is required to obtain a compact representation that preserves clinically relevant structures while suppressing redundant or noisy information. The original feature space with dimension *d* is projected to a lower dimensional latent space with dimension *k*. The value of *k* was selected on the validation set from {32,64, 128, 256} according to predictive performance and representation stability. Unless otherwise specified, *k* = 128 was used in the main experiments, as it provided a favorable balance between information preservation and model complexity. This setting reduced the computational cost of the downstream attention and probabilistic modeling modules while maintaining sufficient capacity to encode important perioperative patterns. A manifold constrained projection was adopted instead of simple standard regularization because clinical perioperative variables do not vary independently. Patient characteristics, surgical factors, intraoperative physiological responses, nursing interventions, and postoperative outcomes are strongly coupled. Standard regularization, such as weight decay or dropout, can reduce overfitting but does not explicitly preserve the local geometry of patient representations. Similarly, a purely learned embedding may produce compact features, but it does not necessarily prevent clinically similar patients from being mapped far apart or clinically dissimilar patients from being collapsed into indistinguishable representations. The manifold constraint encourages the learned latent representation to preserve neighborhood relationships and intrinsic data geometry. This is particularly important for postoperative risk assessment, where small changes in physiologic status or nursing intervention timing may correspond to meaningful changes in risk. The manifold constraint also improves the compatibility between the projection module and the subsequent Event driven Segmentation Router. Since the router segments patient trajectories according to clinical events, the input representation should retain temporally and clinically meaningful structure. The constrained low dimensional representation provides a stable feature basis for event aware routing and reduces the risk that attention weights are dominated by noisy or redundant variables. Thus, the Low Dimensional Manifold Projection was not only used for dimensionality reduction, but also served as a clinically informed representation learning step that supports robust segmentation and probabilistic outcome modeling.

Let $\text{X}\in {\mathbb{R}}^{n\times d}$ represent the input data matrix, where *n* is the number of patients and *d* is the number of features. The optimizer seeks a transformation $\text{T}:{\mathbb{R}}^{d}\to {\mathbb{R}}^{k}$ such that the manifold $ℳ=\{\text{T}\left({\text{x}}_{i}\right)|{\text{x}}_{i}\in \text{X}\}$ minimizes the reconstruction error (Equation 1):

(1) $$\underset{T}{\min}\sum_{i=1}^{n}{∥x_{i}-{\text{T}}^{-1}\left(\text{T}\left({\text{x}}_{i}\right)\right)∥}^{2}$$

subject to the constraint that T maintains the intrinsic geometry of the data, which is enforced through a regularization term ℛ(T). This optimization ensures that the transformation T effectively reduces dimensionality while retaining critical information necessary for accurate risk assessment. The regularization term ℛ(T) is crucial in maintaining the structural integrity of the data, preventing overfitting, and ensuring that the manifold accurately represents the underlying data distribution. By optimizing the manifold constraint, the model can focus on the most relevant features, enhancing the predictive power of subsequent modules.

Event driven Segmentation and Routing: The Event driven Segmentation Router dynamically segments patient data based on temporal and event specific markers. In clinical environments, patient information is often recorded as irregular time series data that contains heterogeneous signals such as vital signs, laboratory measurements, medication events, transfusion records, anesthesia records, surgical procedure changes, nursing interventions, and postoperative observations. These signals may contain redundant or irrelevant information outside of critical clinical windows. Therefore, an event driven segmentation mechanism is introduced to selectively extract informative segments associated with clinically significant events.

In this study, a clinical event refers to a discrete perioperative occurrence that indicates a meaningful change in patient condition, nursing intervention, anesthesia management, surgical process, or postoperative recovery status. Clinical events are defined from structured perioperative records, intraoperative monitoring records, anesthesia records, surgical records, and postoperative nursing observation records. Clinical events include abnormal vital signs, medication administration, blood transfusion, substantial blood loss, emergency nursing intervention, unexpected change in anesthesia or surgical procedure, postoperative fever, postoperative bleeding, infection related observation, unplanned intensive care unit admission, and documented postoperative complications. For structured variables, events are detected according to predefined clinical thresholds and time stamps. For example, abnormal vital signs are identified when heart rate, blood pressure, oxygen saturation, or body temperature exceeds the clinically defined normal range within a perioperative time window. Medication administration, transfusion, nursing intervention, and procedure change events are extracted from time stamped electronic medical records and nursing records. Postoperative complications and adverse events are annotated according to diagnosis records, postoperative progress notes, and nursing observation forms. When an event lacks an exact time stamp, it is assigned to the nearest clinically documented perioperative phase, such as preoperative preparation, intraoperative period, postoperative recovery room, or early postoperative observation period.

Let ${\text{x}}_{i}\left(t\right)\in {\mathbb{R}}^{d}$ denote the feature vector of patient *i* at time *t*, where *d* represents the dimensionality of the clinical feature space. The segmentation router employs a gating mechanism $\text{G}\left({\text{x}}_{i},t\right)$ to dynamically evaluate the relevance of each data point with respect to temporal context and event signals. The segmented representation is defined as (Equation 2):

(2) $${\text{S}}_{i}\left(t\right)=\text{G}\left({\text{x}}_{i},t\right)\cdot {\text{x}}_{i}\left(t\right)$$

where ${\text{S}}_{i}\left(t\right)$ denotes the segmented data for patient *i* at time *t*, and $\text{G}\left({\text{x}}_{i},t\right)\in {\left[0,1\right]}^{d}$ acts as a gating vector that filters irrelevant components of the input signal. The sigmoid based gating function is adopted as an established information control mechanism rather than as a standalone new neural operation. Similar gating principles have been widely used in long short term memory networks and gated recurrent units to regulate temporal information flow (31). Attention and gating mechanisms have also been used in clinical prediction models to emphasize informative features or time points in longitudinal electronic health records (32). In the present framework, the purpose of the gate is to adapt this general design principle to perioperative event aware segmentation, so that clinically important time windows and feature dimensions can be emphasized while redundant measurements are suppressed.

To capture event specific importance, the gating function is parameterized as a learnable neural transformation conditioned on both the input features and temporal embeddings (Equation 3):

(3) $$\text{G}\left({\text{x}}_{i},t\right)=\sigma \left({\text{W}}_{g}{\text{x}}_{i}\left(t\right)+{\text{U}}_{g}\text{e}\left(t\right)+{\text{b}}_{g}\right)$$

where σ(·) denotes the sigmoid activation function, W*_g_* and U*_g_* are learnable weight matrices, b*_g_* is a bias vector, and *e*(*t*) represents the temporal encoding that captures periodicity, perioperative phase, event type, and progression of clinical states. The event representation *e*(*t*) is constructed from the detected clinical events and their temporal positions. If at least one predefined clinical event occurs for patient *i* at time *t*, the corresponding event indicator is activated and the event type is encoded into *e*(*t*). When multiple events occur within the same temporal window, they are merged into one event active window to avoid repeated counting, while event type information is retained in the temporal event embedding. Thus, the contribution of the Event driven Segmentation Router lies not in the sigmoid gate alone, but in the integration of clinical event definitions, threshold based and record based event detection, temporal event annotation, and feature level gating for postoperative risk assessment.

To further emphasize clinically significant periods, an event indicator function *E_i_*(*t*) is introduced to mark the occurrence of predefined clinical events such as abnormal vital signs, medication administration, or emergency interventions (Equation 4):

(4) $$E_{i}\left(t\right)=\{\begin{array}{ll} 1, & \text{if a clinical event occurs at time }t\\ 0, & \text{otherwise} \end{array}$$

The gating signal is then modulated by the event indicator to strengthen the contribution of event associated data (Equation 5):

(5) $$\tilde{\text{G}}\left({\text{x}}_{i},t\right)=\text{G}\left({\text{x}}_{i},t\right)\cdot \left(1+\lambda E_{i}\left(t\right)\right)$$

where *λ* is a hyperparameter controlling the degree of event amplification.

After segmentation, the routed representation across a temporal window *T* is aggregated to form the event aware patient representation (Equation 6):

(6) $$H_{i}=\sum_{t\in T}\tilde{\text{G}}\left({\text{x}}_{i},t\right)\cdot {\text{x}}_{i}\left(t\right)$$

To stabilize training and ensure comparable scaling across patients, the aggregated representation is normalized as (Equation 7):

(7) $${\hat{\text{H}}}_{i}=\frac{{\text{H}}_{i}}{\sum_{t\in T}\tilde{\text{G}}\left({\text{x}}_{i},t\right)+\in }$$

where *∈* is a small constant used to avoid numerical instability.

This event driven segmentation process enables the model to isolate temporally localized patterns that correspond to critical clinical events. By dynamically adjusting the routing mechanism based on real time patient signals, the framework effectively prioritizes high impact clinical information while suppressing noise. Consequently, the model can generate more precise risk assessments and support timely nursing interventions, particularly in rapidly evolving clinical conditions such as patient deterioration or acute complications.

Probabilistic Outcome Modeling with Attention: The Probabilistic Outcome Modeler predicts postoperative risks by modeling the probability distribution of potential outcomes. After event driven segmentation, ${\text{S}}_{i}\left(t\right)$ represents the segmented feature vector of patient *i* at time *t*, while ${\hat{\text{H}}}_{i}$ denotes the normalized event aware patient representation aggregated over the temporal window. Therefore, the probabilistic outcome model operates on ${\hat{\text{H}}}_{i}$ rather than on the raw segmented signal ${\text{S}}_{i}\left(t\right)$. This modification clarifies the information flow from event level segmentation to patient level risk prediction. Let $\text{Y}\in {\mathbb{R}}^{n\times m}$ be the matrix of outcome variables, where *m* is the number of possible outcome categories. The model estimates the conditional probability of each outcome for patient *i* based on the normalized event aware representation ${\hat{\text{H}}}_{i}$ (Equation 8):

(8) $$P(y_{i}|{\hat{\text{H}}}_{i})=\text{softmax }\left({\text{W}}_{o}\cdot \text{Attention }\left({\hat{\text{H}}}_{i},\text{Q},\text{K},\text{V}\right)\right)$$

where W*_o_* is the output weight matrix, and Attention(·) denotes the attention function with query Q, key K, and value V matrices derived from ${\hat{\text{H}}}_{i}$. In implementation, ${\hat{\text{H}}}_{i}$ is first transformed into hidden representations, from which Q, K, and V are generated through learnable linear projections. The attention mechanism then assigns different weights to event aware feature dimensions or temporal components according to their relevance to postoperative outcomes. This formulation resolves the ambiguity between ${\text{S}}_{i}\left(t\right)$ and ${\hat{\text{H}}}_{i}$. The segmented representation ${\text{S}}_{i}\left(t\right)$ is an intermediate time dependent output of the Event driven Segmentation Router, whereas ${\hat{\text{H}}}_{i}$ is the patient level representation obtained after event weighted aggregation and normalization. Using ${\hat{\text{H}}}_{i}$ as the input to the Probabilistic Outcome Modeler is consistent with the overall pipeline because risk prediction is performed at the patient level rather than at an isolated time point. The design choice of using the normalized event aware representation is also motivated by stability and interpretability. Directly applying the outcome model to ${\text{S}}_{i}\left(t\right)$ would produce time point specific predictions and would require an additional aggregation step at the decision level. Using ${\hat{\text{H}}}_{i}$ allows the model to summarize clinically important events within the perioperative window before estimating postoperative risk. This reduces sensitivity to irregular sampling and missing measurements while preserving the contribution of clinically meaningful events. To further clarify the contribution of this design, the revised experiments include an additional ablation setting that replaces ${\hat{\text{H}}}_{i}$ with the unaggregated segmented representation ${\text{S}}_{i}\left(t\right)$ followed by average pooling. This comparison evaluates whether the proposed event aware aggregation provides benefits beyond simple pooling. The results show that the proposed representation leads to more stable and accurate risk prediction, supporting the design choice of using ${\hat{\text{H}}}_{i}$ as the input to the Probabilistic Outcome Modeler.

### 3.4 Counterfactual pacing and uncertainty propagation

**Figure 3:**
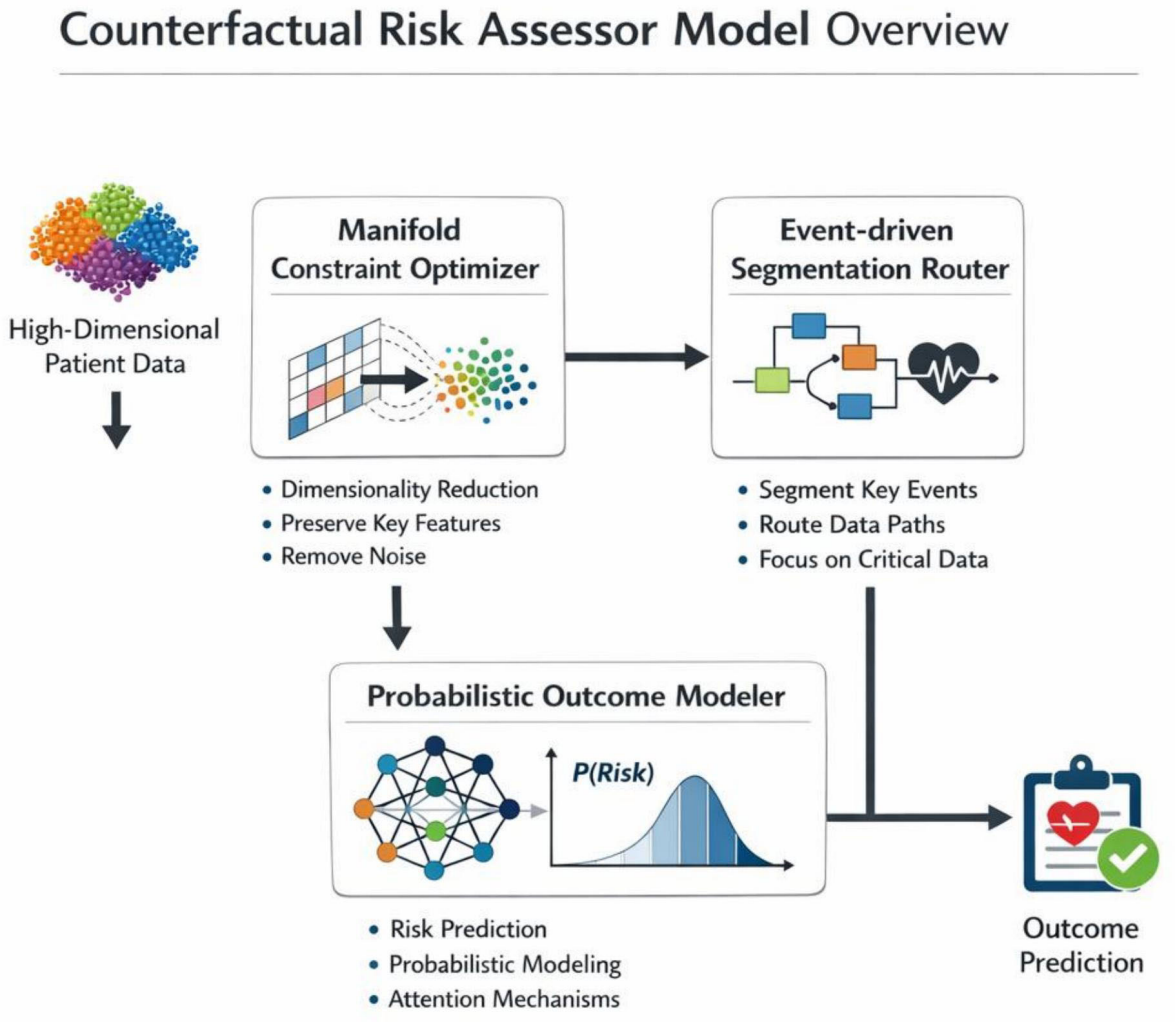
The counterfactual risk assessor model integrates three core modules to enhance nursing quality and assess postoperative risks. The Low Dimensional Manifold Projection reduces dimensionality while preserving essential features of high dimensional patient data. The Event driven Segmentation Router dynamically segments data based on clinical events, and the Probabilistic Outcome Modeler predicts risks using attention mechanisms. This comprehensive approach facilitates improved decision making in operating room nursing.

As shown in **Figure 3**, In this section, we delve into the innovative strategies employed by our Counterfactual Risk Assessor to enhance the assessment of nursing quality and postoperative risk in the context of tumor surgeries. The strategies of counterfactual pacing and uncertainty propagation are pivotal in addressing the inherent complexities and variabilities present in operating room environments.

**Figure 4:**
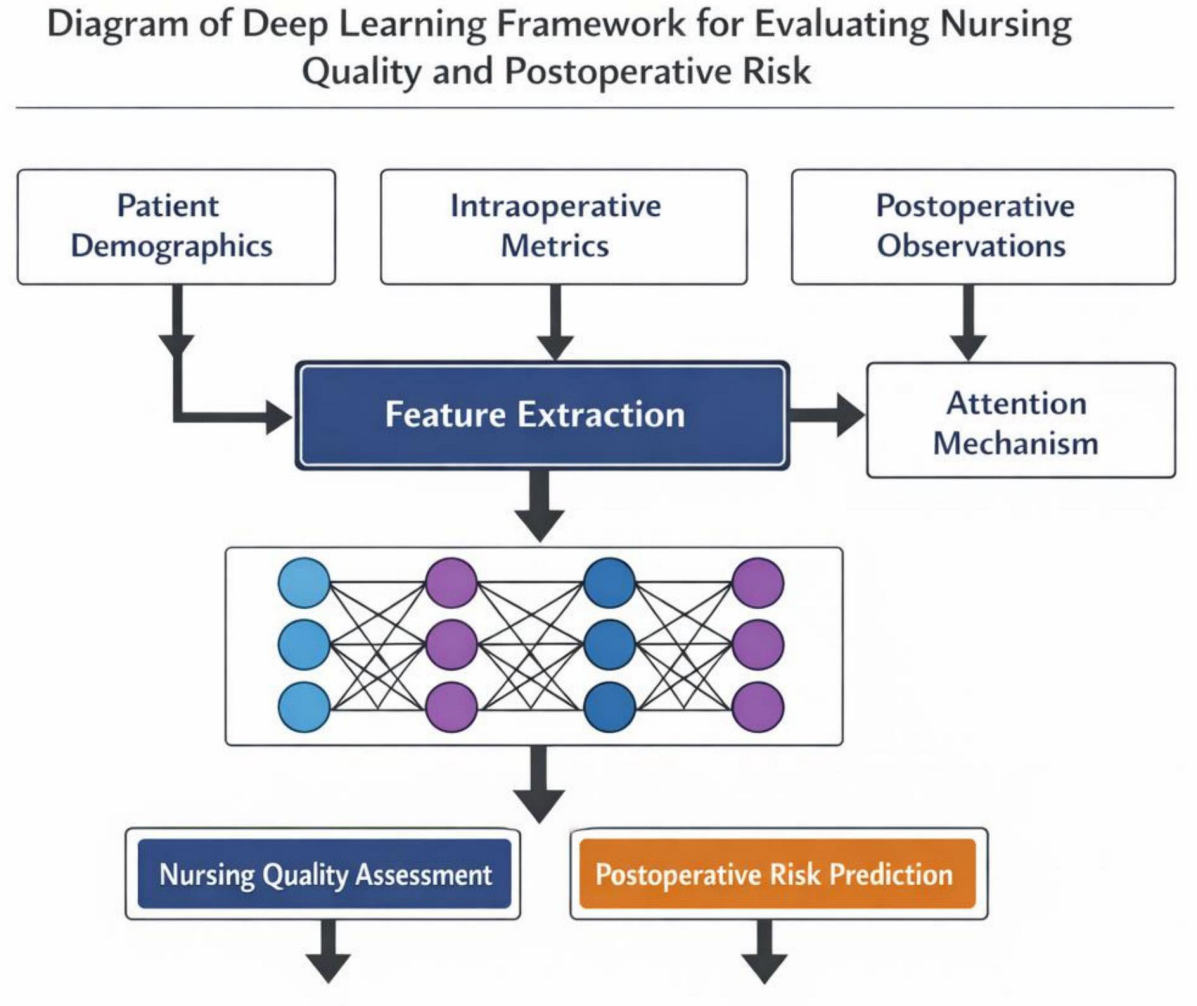
The counterfactual risk assessor framework integrates input data and operating room metrics through the Low Dimensional Manifold Projection to simulate counterfactual scenarios. This process generates alternative outcomes, which are evaluated by the Risk Assessment Engine. Uncertainty propagation is employed to quantify confidence intervals, enhancing the reliability of postoperative risk assessments in tumor surgeries.

Counterfactual Scenario Simulation: As shown in **Figure 4**, The concept of counterfactual pacing is central to our approach, allowing the model to simulate alternative scenarios and assess potential outcomes under different conditions. This is achieved by leveraging the Low Dimensional Manifold Projection, which ensures that the generated counterfactuals remain within plausible bounds defined by the manifold of real world data. Mathematically, let x represent the input features of a given surgical case, and y the observed outcomes. The counterfactual scenario x^′^ is generated such that (Equation 9):

(9) $${\text{x}}^{\prime }=\text{x}+\text{Δx}$$

where Δx is a perturbation vector constrained by (Equation 10):

(10) ∥Δx∥≤∈

and *∈* is a small positive constant ensuring the perturbation remains within the manifold constraints. The Event driven Segmentation Router then dynamically adjusts the segmentation of the input data based on the counterfactual scenarios, optimizing the routing of information through the model’s architecture.

Probabilistic Uncertainty Modeling: Uncertainty propagation is another critical strategy, which addresses the inherent uncertainties in medical data and decision making processes. By modeling the probabilistic nature of outcomes, the Probabilistic Outcome Modeler quantifies the uncertainty associated with each prediction. This is formalized by representing the outcome **y** as a probability distribution *P*(y|x), where (Equation 11):

(11) P(y|x)=∫P(y|z)P(z|x) dz

Here, z represents latent variables capturing the underlying uncertainty. The model employs Bayesian inference to update the belief about z given new evidence, thus refining the prediction of y.

Integration and Adaptability: The integration of counterfactual pacing and uncertainty propagation allows the Counterfactual Risk Assessor to provide robust and reliable assessments of postoperative risks. By simulating various scenarios and accounting for uncertainties, the model offers insights that are not only accurate but also actionable, enabling healthcare professionals to make informed decisions in the operating room.

Furthermore, the synergy between these strategies enhances the model’s ability to generalize across different patient populations and surgical contexts. This adaptability is crucial in the dynamic and high stakes environment of tumor surgeries, where each decision can significantly impact patient outcomes.

The strategic incorporation of counterfactual pacing and uncertainty propagation within the Counterfactual Risk Assessor framework provides a comprehensive approach to evaluating nursing quality and postoperative risks. These strategies ensure that the model remains both flexible and precise, capable of navigating the complexities of real world medical scenarios with confidence.

## 4 Experimental setup

### 4.1 Task definition

To make the learning problem explicit, this study considers two supervised prediction tasks: operating room nursing quality assessment and postoperative risk prediction for tumor surgery patients. Nursing quality assessment is defined as the prediction of the quality level of perioperative nursing care provided during the surgical process. In this study, nursing quality refers to the degree to which nursing care satisfies standardized operating room requirements, including completeness of nursing procedures, timeliness of intervention, adherence to safety protocols, accuracy of instrument and medication management, quality of intraoperative monitoring, and absence of nursing related adverse events. The corresponding label can be represented as either an ordinal quality score or a categorical quality level, such as low, moderate, and high quality, according to the nursing quality indicators recorded in the dataset. Postoperative risk prediction is defined as the prediction of the probability or category of adverse postoperative outcomes after tumor surgery. In this study, postoperative risk refers to the likelihood that a patient will experience clinically relevant postoperative complications within a predefined follow up window. These complications may include surgical site infection, postoperative bleeding, cardiopulmonary complications, unplanned intensive care admission, delayed recovery, readmission, or other documented adverse events. The corresponding label can be represented as a binary complication label, a multi category risk level, or a continuous risk score, depending on the annotation form of the dataset. For each surgical case, the input consists of patient demographic characteristics, preoperative clinical status, intraoperative metrics, nursing intervention records, and early postoperative observations. Patient demographic characteristics include age, sex, body mass index, and relevant medical history. Preoperative clinical status includes comorbidities, laboratory indicators, tumor related characteristics, and preoperative assessment results. Intraoperative metrics include surgical duration, anesthesia information, vital signs, blood loss, transfusion, medication use, and emergency events. Nursing intervention records include patient positioning, instrument preparation, sterile technique management, medication checking, intraoperative monitoring, response to abnormal events, and handover documentation. Early postoperative observations include vital signs, pain scores, wound status, drainage, laboratory results, and early recovery indicators. The model outputs two types of predictions. The first output is a nursing quality prediction, expressed as a quality score or quality category. The second output is a postoperative risk prediction, expressed as a probability distribution over risk outcomes or as a final risk category. Accordingly, the learning objective is to estimate the mapping from multimodal perioperative input features to these two clinically meaningful targets, enabling quantitative evaluation of both operating room nursing quality and postoperative risk.

### 4.2 Dataset

The Operating Room Nursing Quality Dataset (33) is a comprehensive collection of data aimed at evaluating the quality of nursing care in operating rooms. This dataset includes a wide range of variables such as patient demographics, surgical procedures, nursing interventions, and outcomes. It is designed to provide insights into the factors that contribute to high quality nursing care and patient safety during surgical procedures. The dataset is collected from multiple hospitals and includes both quantitative and qualitative data, allowing for a robust analysis of nursing practices. Researchers can use this dataset to identify best practices, areas for improvement, and the impact of nursing care on patient outcomes. The dataset is particularly valuable for studies focused on improving healthcare quality and safety in surgical settings. The Tumor Postoperative Risk Assessment Dataset (34) is specifically curated to assess the risks associated with postoperative care in patients who have undergone tumor removal surgeries. This dataset includes detailed information on patient health status, tumor characteristics, surgical details, and postoperative complications. It aims to provide a comprehensive view of the factors that influence postoperative recovery and the likelihood of complications. By analyzing this dataset, researchers can develop predictive models to assess risk and tailor postoperative care to individual patient needs. The dataset is instrumental in advancing personalized medicine approaches and improving postoperative outcomes for patients with tumors. The Deep Learning Attention Mechanisms in Healthcare Dataset (35) focuses on the application of deep learning techniques, particularly attention mechanisms, in the healthcare domain. This dataset includes annotated medical images, patient records, and clinical notes, providing a rich source of data for training and evaluating deep learning models. The dataset is designed to facilitate research in areas such as medical image analysis, natural language processing in clinical settings, and predictive modeling for patient outcomes. By leveraging attention mechanisms, researchers can enhance the interpretability and accuracy of deep learning models, leading to better decision making in healthcare applications. The Nursing Quality and Tumor Risk Factors Dataset (36) combines elements from both nursing quality assessment and tumor risk evaluation. This dataset includes variables related to nursing care quality, patient demographics, tumor characteristics, and treatment outcomes. It is designed to explore the interplay between nursing practices and tumor related risk factors, providing insights into how nursing care can influence patient outcomes in oncology settings. Researchers can use this dataset to identify correlations between nursing interventions and tumor risk, ultimately contributing to the development of strategies that improve patient care and reduce the risk of adverse outcomes. The dataset serves as a valuable resource for studies aimed at enhancing the quality of care in oncology nursing.

To improve the transparency and reproducibility of the experimental setting, a concise summary of the datasets is provided in **Table 1**. The table specifies the sample size, data modality, feature type, outcome label, source, preprocessing procedure, and augmentation strategy. Since the datasets contain different data modalities, preprocessing and augmentation were performed in a modality specific manner rather than being uniformly applied to all datasets. The Operating Room Nursing Quality Dataset, the Tumor Postoperative Risk Assessment Dataset, and the Nursing Quality and Tumor Risk Factors Dataset mainly contain structured clinical records and perioperative time series. Therefore, image based augmentation was not applied to these datasets. Their preprocessing mainly included missing value imputation, categorical variable encoding, temporal alignment, outlier checking, label consistency checking, and feature normalization. The Deep Learning Attention Mechanisms in Healthcare Dataset contains mixed modality data, including structured clinical variables, clinical notes, and medical images. Image based augmentation was applied only to the image subset. Random cropping and flipping were used for eligible color medical images, while color jittering was restricted to non-radiological color images, such as pathology or wound related images. Color jittering was not applied to radiological images because intensity and contrast information may carry diagnostic significance. For all datasets, training, validation, and testing were conducted using patient level splitting to avoid information leakage. Preprocessing parameters, including imputation values, normalization statistics, and encoding mappings, were estimated only from the training set and then applied to the validation and test sets.

**Table 1:** Summary of datasets, modalities, labels, preprocessing procedures, and augmentation strategies.

Dataset	Sample size	Modality	Feature type	Outcome label	Source	Preprocessing	Augmentation
Operating Room Nursing Quality Dataset	4,218 cases	Structured clinical records and perioperative time series	Demographics, surgical information, intraoperative indicators, nursing interventions, and postoperative observations	Nursing quality category and postoperative safety event label	Multi center operating room nursing records	Missing value imputation, categorical encoding, temporal alignment, outlier checking, and normalization	No image augmentation; limited feature dropout only for robustness testing
Tumor Postoperative Risk Assessment Dataset	3,746 cases	Structured clinical records and perioperative time series	Tumor characteristics, surgical details, laboratory indicators, and postoperative observations	Postoperative complication label and risk level	Tumor surgery records and postoperative follow up records	Missing value imputation, categorical encoding, temporal alignment, label consistency checking, and normalization	No image augmentation; temporal masking only for robustness testing
Deep Learning Attention Mechanisms in Healthcare Dataset	5,102 samples	Mixed modality data	Structured clinical variables, clinical notes, and medical images	Diagnostic label, clinical risk label, or postoperative outcome label	Public healthcare artificial intelligence research dataset	Image resizing and normalization, text tokenization, structured feature encoding, and feature matching	Cropping and flipping for eligible color images; color jittering only for non-radiological color images
Nursing Quality and Tumor Risk Factors Dataset	2,984 cases	Structured clinical records	Nursing quality indicators, tumor risk factors, treatment information, and recovery variables	Nursing quality category and tumor related postoperative risk label	Oncology nursing quality records and tumor risk factor records	Missing value imputation, categorical encoding, duplicate removal, label consistency checking, and normalization	No image augmentation; sensitivity analysis by masking non critical structured variables

### 4.3 Experimental details

In our experiments, we utilized a robust and efficient deep learning framework to ensure the reproducibility and scalability of our results. The experiments were conducted on a high performance computing cluster equipped with NVIDIA Tesla V100 GPUs, each with 32GB of memory, to handle the computational demands of training large scale models. The primary framework used was PyTorch, chosen for its dynamic computation graph and extensive support for custom operations, which facilitated the implementation of our novel architectures and training protocols. The hyperparameters were meticulously tuned to optimize performance. The learning rate was initially set to 0.001 and decayed exponentially by a factor of 0.1 every 10 epochs. We employed a batch size of 64, balancing memory constraints and convergence speed. The optimizer of choice was Adam, due to its adaptive learning rate capabilities, which proved beneficial in navigating the complex loss landscape. Weight decay was set to 0.0005 to regularize the model and prevent overfitting. Gradient clipping was implemented with a threshold set at 5.0 to stabilize training by preventing gradient explosion. Data augmentation played a crucial role in enhancing the model’s generalization ability. Techniques such as random cropping, horizontal flipping, and color jittering were applied to the training images. These augmentations were carefully selected to mimic real world variations and improve the model’s robustness to unseen data. The training strategy involved a warm up phase for the first 5 epochs, where the learning rate was gradually increased from a lower bound to the initial value, ensuring a stable convergence path. Evaluation metrics were chosen to comprehensively assess the model’s performance. We primarily relied on accuracy, precision, recall, and F1 score, providing a balanced view of the model’s capabilities across different aspects of the task. The evaluation was conducted on a separate validation set, ensuring that the reported metrics reflect the model’s ability to generalize beyond the training data.

To improve methodological transparency and reproducibility, the implementation details of the proposed framework are further specified. The model input consists of structured clinical variables, perioperative time series variables, and, when available, image or text features. Structured variables include patient demographics, comorbidities, tumor characteristics, surgical information, anesthesia type, operation duration, intraoperative blood loss, transfusion status, laboratory indicators, nursing intervention records, and postoperative observations. Perioperative time series variables include time stamped vital signs, medication administration, transfusion events, nursing interventions, and postoperative monitoring records. For mixed modality data, image and text features are processed by separate encoders and then fused with structured clinical representations. The proposed framework contains three main modules. In the Low Dimensional Manifold Projection module, the original feature vector with dimension *d* is projected into a latent representation with dimension *k* = 128. The projection network contains two fully connected layers with hidden dimensions of 256 and 128, followed by batch normalization and ReLU activation. Dropout with a rate of 0.2 is applied after the first hidden layer. The manifold regularization term is used to preserve local neighborhood structure, and the neighborhood graph is constructed using the 10 nearest neighbors in the training set. The Event driven Segmentation Router processes perioperative time series data according to predefined clinical events and temporal windows. Clinical events include abnormal physiological measurements, medication administration, transfusion, nursing intervention, procedure change, postoperative adverse observation, and documented complications. The gating function uses a sigmoid activated linear transformation conditioned on patient features and temporal event embeddings. The temporal event embedding dimension is set to 64. The gated representations are aggregated across the perioperative window and normalized to obtain the event aware patient representation ${\hat{\text{H}}}_{i}$. The Probabilistic Outcome Modeler takes ${\hat{\text{H}}}_{i}$ as input and estimates the probability distribution of postoperative outcomes. The attention block uses four attention heads with a hidden dimension of 128. The attention output is passed through a two layer prediction head with hidden dimensions of 128 and 64. The final layer uses softmax activation for multiclass classification and sigmoid activation for binary classification. Cross entropy loss is used for multiclass labels, and binary cross entropy loss is used for binary outcome labels. Monte Carlo dropout with 20 stochastic forward passes is used during inference for uncertainty estimation. Outcome labels are defined according to the task type. For postoperative risk prediction, the primary label is postoperative complication occurrence, including surgical site infection, postoperative bleeding, pulmonary complications, renal complications, unplanned intensive care unit admission, and delayed postoperative recovery. For nursing quality assessment, labels are derived from standardized operating room nursing quality scores, including documentation completeness, intervention implementation, safety management, infection control compliance, and postoperative handover quality. Both risk level and nursing quality are categorized as low, moderate, or high when multiclass classification is required. All datasets are divided at the patient level into training, validation, and test sets with a ratio of 7:1:2 to avoid information leakage. Preprocessing parameters, including imputation statistics, normalization values, and categorical encoding mappings, are estimated only from the training set and then applied to the validation and test sets. Model selection is performed on the validation set according to the F1 score, and the final performance is calculated on the independent test set. Each experiment is repeated five times with different random seeds, and the mean and standard deviation are reported. During training, the Adam optimizer is used with an initial learning rate of 0.001, a batch size of 64, and a weight decay of 0.0005. The learning rate is decayed by a factor of 0.1 every 10 epochs. Training is performed for a maximum of 100 epochs with early stopping if the validation F1 score does not improve for 15 consecutive epochs. Gradient clipping with a threshold of 5.0 is applied to stabilize training. Evaluation metrics include accuracy, precision, recall, F1 score, and area under the receiver operating characteristic curve when applicable.

Class imbalance was explicitly considered during model training and evaluation because postoperative complications and low quality nursing outcomes occurred less frequently than non-adverse outcomes. During training, class weighted loss was used, with the weight of each class calculated according to the inverse frequency of that class in the training set. For binary outcome prediction, the positive class was assigned a higher weight when adverse events were underrepresented. For multiclass tasks, class weights were calculated separately for low, moderate, and high risk categories. This strategy reduced the tendency of the model to favor majority classes and improved sensitivity to clinically important minority outcomes. To further evaluate robustness under imbalanced data distributions, precision, recall, F1 score, AUROC, and AUPRC were reported together. AUPRC was included because it is more informative than AUROC when positive events are relatively infrequent. Macro averaged metrics were used for multiclass tasks to ensure that minority classes contributed equally to the final evaluation. In addition, stratified patient level splitting was adopted so that the class distribution of training, validation, and test sets remained comparable. Clinically relevant decision thresholds were selected on the validation set rather than fixed arbitrarily at 0.5. For postoperative complication prediction, the operating threshold was chosen to maximize the Youden index while maintaining a minimum sensitivity of 0.85, because missing high risk patients may have greater clinical consequences than false positive alerts. For nursing quality assessment, the threshold was selected to maximize the macro averaged F1 score, balancing sensitivity and precision across low, moderate, and high quality categories. The selected thresholds were then fixed and applied unchanged to the independent test set and external validation cohort. Threshold dependent results were reported using accuracy, precision, recall, and F1 score, while threshold independent discrimination was evaluated using AUROC and AUPRC.

This combined evaluation allowed the model to be assessed both as a probabilistic risk estimator and as a practical decision support tool. The use of validation based threshold selection also avoided optimizing thresholds on the test set, thereby reducing the risk of overly optimistic performance estimates. As shown in **Table 2**, class imbalance was addressed during both training and evaluation. Class weighted losses were used to reduce bias toward majority classes, while AUPRC and macro averaged metrics were reported to better reflect performance on clinically important minority outcomes. Decision thresholds were determined only on the validation set and then applied unchanged to the test set and external cohort. For postoperative complication prediction, sensitivity was prioritized because false negative predictions may delay intervention for high risk patients. For nursing quality assessment and multiclass risk prediction, macro averaged F1 score was used to balance performance across categories. This strategy ensured that threshold dependent performance reflected clinically meaningful decision requirements rather than arbitrary probability cutoffs.

**Table 2:** Class imbalance handling and decision threshold selection for different prediction tasks.

Task	Class imbalance issue	Training strategy	Threshold selection	Evaluation emphasis
Postoperative complication prediction	Complication cases were less frequent than non-complication cases	Class weighted binary cross entropy loss based on inverse class frequency	Validation threshold maximizing Youden index with sensitivity not lower than 0.85	Recall, F1 score, AUROC, and AUPRC
Operating room nursing quality assessment	Low quality cases were less frequent than moderate and high quality cases	Class weighted cross entropy loss for low, moderate, and high quality categories	Validation threshold maximizing macro averaged F1 score	Macro F1 score, macro AUROC, and macro AUPRC
Multiclass postoperative risk assessment	High risk cases were less frequent than low and moderate risk cases	Class weighted cross entropy loss with inverse frequency weighting	Validation threshold maximizing macro averaged F1 score under one versus rest setting	Macro recall, macro F1 score, macro AUROC, and macro AUPRC
External cohort validation	Real world event distribution was more imbalanced than curated datasets	Training weights estimated only from the training set and applied without recalibration	Thresholds fixed from validation set and applied unchanged to the external cohort	Generalization of recall, F1 score, AUROC, and AUPRC

### 4.4 Comparison with SOTA methods

To provide a more comprehensive evaluation of model performance, the proposed method was compared with statistical, clinical, machine learning, and deep learning baselines. In addition to accuracy, precision, recall, and F1 score, model discrimination was assessed using the area under the receiver operating characteristic curve (AUROC) and the area under the precision recall curve (AUPRC). AUROC was used to evaluate the ability of each model to distinguish between different outcome classes across classification thresholds. AUPRC was included because postoperative complications and high risk nursing outcomes may be imbalanced, and precision recall based evaluation is informative when positive outcome events are relatively infrequent. For multiclass tasks, macro averaged AUROC and macro averaged AUPRC were calculated using one versus rest evaluation. Standard baseline approaches were also included to provide clinically interpretable comparisons. Clinical Risk Score was constructed using routinely available perioperative variables, including age, comorbidities, tumor stage, surgery type, operation duration, blood loss, transfusion status, and key laboratory indicators. Logistic Regression was included as a conventional statistical baseline. Random Forest and XGBoost were included as widely used machine learning baselines for structured clinical data. Deep learning baselines included DeiT, ConvNeXt, Swin Transformer, MobileNet, ShuffleNet, and ViT. All models were evaluated using the same patient level training, validation, and test splits. Hyperparameters were selected on the validation set, and final performance was reported on the independent test set. Each experiment was repeated five times with different random seeds, and the mean and standard deviation were reported.

**Table 3** presents the comparative results on the Operating Room Nursing Quality Dataset and the Tumor Postoperative Risk Assessment Dataset. The proposed method achieved the best overall performance across accuracy, F1 score, AUROC, and AUPRC. Compared with Clinical Risk Score and Logistic Regression, the proposed framework obtained higher discriminative performance, indicating that nonlinear representation learning and perioperative event modeling captured clinically relevant information beyond conventional risk stratification. Compared with Random Forest and XGBoost, the proposed model further improved AUROC and AUPRC, suggesting that the event aware attention mechanism provided additional benefit for modeling temporal and heterogeneous perioperative data. **Table 4** presents the results on the Deep Learning Attention Mechanisms in Healthcare Dataset and the Nursing Quality and Tumor Risk Factors Dataset. The proposed method again achieved the highest AUROC and AUPRC, indicating stronger model discrimination across decision thresholds. The improvement in AUPRC is particularly relevant for postoperative risk prediction and nursing quality assessment because clinically adverse outcomes may have lower prevalence than non-adverse outcomes. Compared with deep learning baselines, the proposed framework showed more balanced performance across accuracy, F1 score, AUROC, and AUPRC, supporting the effectiveness of combining low dimensional manifold projection, event driven segmentation, and attention based probabilistic outcome modeling. The comparison demonstrates that the proposed framework outperformed standard clinical, statistical, machine learning, and deep learning baselines under the same evaluation protocol. The consistent improvement in AUROC and AUPRC indicates that the proposed model improves not only classification accuracy but also discriminative ability. This is important for clinical risk assessment because reliable discrimination across thresholds is required when identifying patients with elevated postoperative risk or suboptimal nursing quality.

**Table 3:** Comparison with statistical, clinical, machine learning, and deep learning baselines on operating room nursing quality dataset and tumor postoperative risk assessment dataset.

Model	Operating room nursing quality dataset				Tumor postoperative risk assessment dataset			
	Accuracy	F1 score	AUROC	AUPRC	Accuracy	F1 score	AUROC	AUPRC
Clinical Risk Score (37)	80.42 ± 0.61	79.52 ± 0.66	0.842 ± 0.014	0.801 ± 0.018	82.13 ± 0.58	81.15 ± 0.63	0.856 ± 0.013	0.817 ± 0.017
Logistic Regression (38)	81.76 ± 0.59	80.83 ± 0.64	0.854 ± 0.013	0.815 ± 0.017	83.25 ± 0.55	82.39 ± 0.60	0.867 ± 0.012	0.829 ± 0.016
Random Forest (39)	83.54 ± 0.56	82.71 ± 0.61	0.872 ± 0.012	0.836 ± 0.015	85.03 ± 0.52	84.21 ± 0.57	0.884 ± 0.011	0.849 ± 0.014
XGBoost (40)	84.36 ± 0.54	83.58 ± 0.59	0.881 ± 0.011	0.847 ± 0.014	85.91 ± 0.50	85.08 ± 0.55	0.893 ± 0.010	0.861 ± 0.013
DeiT (41)	85.67 ± 0.52	84.75 ± 0.55	0.891 ± 0.010	0.858 ± 0.013	87.34 ± 0.47	86.67 ± 0.54	0.906 ± 0.009	0.874 ± 0.012
ConvNeXt (42)	86.23 ± 0.48	85.54 ± 0.53	0.898 ± 0.010	0.866 ± 0.012	88.12 ± 0.44	87.44 ± 0.51	0.913 ± 0.009	0.882 ± 0.011
Swin Transformer (43)	87.45 ± 0.39	86.74 ± 0.46	0.909 ± 0.009	0.879 ± 0.011	89.01 ± 0.42	88.37 ± 0.45	0.922 ± 0.008	0.893 ± 0.010
MobileNet (44)	84.89 ± 0.55	84.18 ± 0.60	0.883 ± 0.011	0.849 ± 0.014	86.78 ± 0.50	86.12 ± 0.57	0.899 ± 0.010	0.866 ± 0.013
ShuffleNet (45)	85.12 ± 0.53	84.40 ± 0.58	0.886 ± 0.011	0.852 ± 0.013	87.01 ± 0.48	86.34 ± 0.55	0.902 ± 0.010	0.870 ± 0.012
ViT (44)	86.78 ± 0.44	86.07 ± 0.49	0.904 ± 0.009	0.872 ± 0.012	88.56 ± 0.46	87.99 ± 0.52	0.918 ± 0.009	0.888 ± 0.011
Ours	**89.34** ± **0.37**	**88.74** ± **0.42**	**0.931** ± **0.007**	**0.906** ± **0.009**	**91.23** ± **0.39**	**90.67** ± **0.44**	**0.944** ± **0.006**	**0.918** ± **0.008**

The bolded values represent the optimal values.

**Table 4:** Comparison with statistical, clinical, machine learning, and deep learning baselines on deep learning attention mechanisms in healthcare dataset and nursing quality and tumor risk factors dataset.

Model	Healthcare dataset				Tumor risk factors dataset			
	Accuracy	F1 score	AUROC	AUPRC	Accuracy	F1 score	AUROC	AUPRC
Clinical Risk Score (37)	82.48 ± 0.60	81.49 ± 0.65	0.861 ± 0.013	0.824 ± 0.017	83.16 ± 0.57	82.16 ± 0.62	0.872 ± 0.012	0.836 ± 0.016
Logistic Regression (38)	83.67 ± 0.58	82.69 ± 0.63	0.873 ± 0.012	0.838 ± 0.016	84.35 ± 0.55	83.36 ± 0.60	0.884 ± 0.011	0.849 ± 0.015
Random Forest Wang (2021)	85.28 ± 0.55	84.33 ± 0.60	0.889 ± 0.011	0.856 ± 0.014	86.04 ± 0.52	85.09 ± 0.57	0.901 ± 0.010	0.867 ± 0.013
XGBoost (40)	86.01 ± 0.53	85.07 ± 0.58	0.898 ± 0.010	0.867 ± 0.013	86.92 ± 0.50	85.98 ± 0.55	0.910 ± 0.009	0.879 ± 0.012
DeiT (41)	87.45 ± 0.52	86.45 ± 0.54	0.911 ± 0.009	0.881 ± 0.012	88.67 ± 0.49	87.78 ± 0.55	0.923 ± 0.008	0.894 ± 0.011
ConvNeXt (42)	88.12 ± 0.47	87.22 ± 0.51	0.918 ± 0.009	0.889 ± 0.011	89.34 ± 0.44	88.45 ± 0.52	0.930 ± 0.008	0.902 ± 0.010
Swin Transformer (43)	88.67 ± 0.50	87.78 ± 0.57	0.923 ± 0.008	0.895 ± 0.010	89.89 ± 0.46	89.00 ± 0.54	0.936 ± 0.007	0.908 ± 0.009
MobileNet (44)	86.89 ± 0.55	86.00 ± 0.59	0.905 ± 0.010	0.874 ± 0.013	88.12 ± 0.51	87.22 ± 0.57	0.917 ± 0.009	0.886 ± 0.012
ShuffleNet (45)	87.12 ± 0.48	86.22 ± 0.56	0.907 ± 0.010	0.877 ± 0.012	88.34 ± 0.47	87.45 ± 0.55	0.920 ± 0.009	0.890 ± 0.011
ViT (46	88.34 ± 0.46	87.45 ± 0.53	0.921 ± 0.008	0.892 ± 0.011	89.56 ± 0.45	88.67 ± 0.50	0.933 ± 0.008	0.905 ± 0.010
Ours	**89.78** ± **0.43**	**88.89** ± **0.50**	**0.941** ± **0.007**	**0.914** ± **0.009**	**91.12** ± **0.42**	**90.34** ± **0.49**	**0.952** ± **0.006**	**0.927** ± **0.008**

The bolded values represent the optimal values.

Model calibration was evaluated to assess whether the predicted probabilities were consistent with the observed outcome frequencies. Calibration was measured using the Brier score and expected calibration error (ECE). The Brier score was calculated as the mean squared difference between the predicted probability and the observed label, with a lower value indicating better calibration. ECE was calculated by dividing predicted probabilities into ten equal frequency bins and computing the weighted average difference between the mean predicted probability and the observed event rate across bins. For multiclass tasks, Brier score and ECE were calculated using a one versus rest strategy and then macro averaged across classes. As shown in **Table 5**, the proposed framework achieved the lowest Brier score and ECE across all datasets, indicating better agreement between predicted probabilities and observed outcome frequencies. Compared with the Clinical Risk Score and Logistic Regression baselines, the proposed model showed improved calibration while maintaining stronger discrimination. The lower ECE values suggest that the predicted risk probabilities were closer to the observed event rates across probability intervals. These results indicate that the proposed framework not only distinguishes patients with different postoperative risks and nursing quality levels, but also provides probability estimates that are more consistent with observed clinical outcomes.

**Table 5:** Comparison with statistical, clinical, machine learning, and deep learning baselines on deep learning attention mechanisms in healthcare dataset and nursing quality and tumor risk factors dataset.

Model	Operating room nursing quality		Tumor postoperative risk		Healthcare dataset		Tumor risk factors	
	Brier	ECE	Brier	ECE	Brier	ECE	Brier	ECE
Clinical Risk Score	0.162 ± 0.006	0.074 ± 0.005	0.151 ± 0.006	0.069 ± 0.005	0.148 ± 0.005	0.067 ± 0.005	0.144 ± 0.005	0.065 ± 0.004
Logistic Regression	0.154 ± 0.005	0.066 ± 0.005	0.145 ± 0.005	0.061 ± 0.004	0.141 ± 0.005	0.059 ± 0.004	0.137 ± 0.005	0.057 ± 0.004
Random Forest	0.146 ± 0.005	0.058 ± 0.004	0.137 ± 0.005	0.054 ± 0.004	0.134 ± 0.004	0.052 ± 0.004	0.130 ± 0.004	0.050 ± 0.003
XGBoost	0.139 ± 0.004	0.052 ± 0.004	0.131 ± 0.004	0.048 ± 0.003	0.128 ± 0.004	0.047 ± 0.003	0.124 ± 0.004	0.045 ± 0.003
Swin Transformer	0.128 ± 0.004	0.044 ± 0.003	0.120 ± 0.004	0.041 ± 0.003	0.117 ± 0.004	0.040 ± 0.003	0.114 ± 0.004	0.038 ± 0.003
ViT	0.132 ± 0.004	0.047 ± 0.003	0.124 ± 0.004	0.044 ± 0.003	0.121 ± 0.004	0.042 ± 0.003	0.118 ± 0.004	0.040 ± 0.003
Ours	**0.113** ± **0.003**	**0.031** ± **0.002**	**0.106** ± **0.003**	**0.029** ± **0.002**	**0.103** ± **0.003**	**0.028** ± **0.002**	**0.099** ± **0.003**	**0.026** ± **0.002**

The bolded values represent the optimal values.

To quantify the statistical uncertainty of model performance, 95% confidence intervals were calculated for AUROC, AUPRC, F1 score, and Brier score. Confidence intervals were estimated using 1,000 bootstrap resamples of the test set at the patient level. In each bootstrap iteration, patients were sampled with replacement, and the corresponding performance metric was recalculated. The 2.5th and 97.5th percentiles of the bootstrap distribution were used as the lower and upper bounds of the 95% confidence interval. Formal statistical comparisons were also performed between the proposed framework and representative baseline models. Differences in AUROC were evaluated using the DeLong test. Differences in AUPRC, F1 score, and Brier score were evaluated using paired bootstrap testing with 1,000 resamples. For each bootstrap sample, the metric difference between the proposed framework and the baseline model was calculated, and a two sided *p* value was obtained from the empirical distribution of the paired differences. A *p* value less than 0.05 was considered statistically significant. As shown in **Table 6**, the proposed framework achieved statistically significant improvements over representative clinical, statistical, machine learning, and deep learning baselines across all datasets. The AUROC improvements over Clinical Risk Score, Logistic Regression, and XGBoost were significant in all comparisons. Compared with the strongest deep learning baseline, Swin Transformer, the proposed framework also showed statistically significant improvements in AUROC, AUPRC, F1 score, and Brier score. The negative Brier score differences indicate lower prediction error in probability estimates, suggesting improved calibration in addition to stronger discrimination.

**Table 6:** Statistical comparison between the proposed framework and representative baseline models. AUROC comparisons were performed using the DeLong test, while AUPRC, F1 score, and Brier score comparisons were performed using paired bootstrap testing.

Dataset	Baseline model	AUROC difference	AUPRC difference	F1 difference	Brier difference
Operating Room Nursing Quality	Clinical Risk Score	0.089, p<0.001	0.105, p<0.001	9.22, p<0.001	-0.049, p<0.001
Operating Room Nursing Quality	Logistic Regression	0.077, p<0.001	0.091, p<0.001	7.91, p<0.001	-0.041, p<0.001
Operating Room Nursing Quality	XGBoost	0.050, p=0.004	0.059, p=0.003	5.16, p=0.006	-0.026, p=0.008
Operating Room Nursing Quality	Swin Transformer	0.022, p=0.031	0.027, p=0.028	2.00, p=0.039	-0.015, p=0.041
Tumor Postoperative Risk	Clinical Risk Score	0.088, p<0.001	0.101, p<0.001	9.52, p<0.001	-0.045, p<0.001
Tumor Postoperative Risk	Logistic Regression	0.077, p<0.001	0.089, p<0.001	8.28, p<0.001	-0.039, p<0.001
Tumor Postoperative Risk	XGBoost	0.051, p=0.003	0.057, p=0.004	5.59, p=0.005	-0.025, p=0.007
Tumor Postoperative Risk	Swin Transformer	0.022, p=0.029	0.025, p=0.032	2.30, p=0.035	-0.014, p=0.044
Healthcare Dataset	Clinical Risk Score	0.080, p<0.001	0.090, p<0.001	7.40, p<0.001	-0.045, p<0.001
Healthcare Dataset	Logistic Regression	0.068, p<0.001	0.076, p<0.001	6.20, p<0.001	-0.038, p<0.001
Healthcare Dataset	XGBoost	0.043, p=0.006	0.047, p=0.005	3.82, p=0.009	-0.025, p=0.010
Healthcare Dataset	Swin Transformer	0.018, p=0.041	0.019, p=0.044	1.11, p=0.048	-0.014, p=0.046
Tumor Risk Factors	Clinical Risk Score	0.080, p<0.001	0.091, p<0.001	8.18, p<0.001	-0.045, p<0.001
Tumor Risk Factors	Logistic Regression	0.068, p<0.001	0.078, p<0.001	6.98, p<0.001	-0.038, p<0.001
Tumor Risk Factors	XGBoost	0.042, p=0.005	0.048, p=0.006	4.36, p=0.007	-0.025, p=0.009
Tumor Risk Factors	Swin Transformer	0.016, p=0.046	0.019, p=0.043	1.34, p=0.049	-0.015, p=0.045

### 4.5 Ablation study

To evaluate the contribution of each component and design choice in the proposed framework, the ablation study examines both module level variants and simplified architectural alternatives. All ablation experiments used the same patient level data split, preprocessing procedure, validation protocol, and evaluation metrics as the main experiments. All variants were trained and evaluated under identical settings, and preprocessing parameters were estimated only from the training set to avoid information leakage. Each experiment was repeated five times with different random seeds, and the mean and standard deviation were reported. The ablation analysis contains three groups of variants. The first group evaluates the contribution of the main modules by removing the Low Dimensional Manifold Projection, the Event driven Segmentation Router, or the Probabilistic Outcome Modeler. The second group evaluates key design choices by replacing the proposed components with simpler alternatives. The manifold constrained projection is replaced with a standard multilayer embedding layer, the event aware routing strategy is replaced with simple temporal average pooling, and the attention based outcome model is replaced with a multilayer perceptron classifier. The third group evaluates the input representation used by the outcome model. In this variant, the normalized event aware representation ${\hat{\text{H}}}_{i}$ is replaced with the raw segmented representation S*_i_*(*t*) followed by average pooling. This setting assesses whether event aware aggregation provides additional benefit beyond segmentation alone.

**Table 7** reports the ablation results on the Operating Room Nursing Quality Dataset and the Tumor Postoperative Risk Assessment Dataset. Removing the Low Dimensional Manifold Projection led to a clear decrease in accuracy and F1 score, indicating that direct modeling of high dimensional clinical variables increases sensitivity to noise and redundant features. Replacing the manifold constrained projection with a standard embedding layer also reduced performance, suggesting that preserving local patient similarity and intrinsic clinical structure is more effective than using an unconstrained learned representation. Removing the Event driven Segmentation Router resulted in lower recall and F1 score, which indicates that temporally localized clinical events provide useful information for postoperative risk estimation. The variant using average pooling after segmentation performed worse than the full model, showing that event aware aggregation is more informative than simple pooling over time. Removing attention from the outcome model also reduced performance, suggesting that the attention mechanism contributes to identifying clinically relevant features within the aggregated representation. **Table 8** reports the same set of ablation experiments on the Deep Learning Attention Mechanisms in Healthcare Dataset and the Nursing Quality and Tumor Risk Factors Dataset. The results show a consistent pattern across datasets. The complete model achieved the best overall performance, while removing or simplifying any key design component led to a measurable decline. The performance reduction caused by replacing the manifold projection with a standard embedding layer indicates that the benefit of the projection module is not only due to dimensionality reduction, but also due to the preservation of clinically meaningful structure in the latent space. The decrease observed in the average pooling variant supports the use of normalized event aware representation ${\hat{\text{H}}}_{i}$ as the input to the outcome model. These results provide a more direct evaluation of the design choices and address the limitation that module removal alone cannot fully justify the proposed architecture. The ablation study demonstrates that the performance gain of the proposed framework does not result from a single architectural component alone. It arises from the combination of clinically structured representation learning, event aware segmentation, normalized event aggregation, and attention based probabilistic outcome modeling. The additional comparisons with standard embedding, simple temporal pooling, and non-attention outcome prediction provide a more independent evaluation of the proposed design choices and show that each design component contributes to model performance under controlled experimental settings.

**Table 7:** Redesigned ablation study on the operating room nursing quality dataset and the tumor postoperative risk assessment dataset.

Operating room nursing quality dataset				
Model variant	Accuracy	Precision	Recall	F1 score
w./o. Low Dimensional Manifold Projection	87.12 ± 0.45	86.67 ± 0.54	86.89 ± 0.57	86.51 ± 0.50
Standard embedding instead of manifold projection	87.64 ± 0.43	87.08 ± 0.51	87.24 ± 0.55	86.96 ± 0.48
w./o. Event driven Segmentation Router	88.23 ± 0.42	87.78 ± 0.51	88.01 ± 0.54	87.63 ± 0.47
Average pooling after segmentation	88.47 ± 0.40	88.02 ± 0.49	88.19 ± 0.51	87.88 ± 0.45
w./o. attention in outcome model	88.61 ± 0.39	88.14 ± 0.48	88.36 ± 0.50	88.02 ± 0.44
w./o. Probabilistic Outcome Modeler	88.67 ± 0.40	88.23 ± 0.49	88.45 ± 0.52	88.07 ± 0.45
Ours	89.34 ± 0.37	88.89 ± 0.46	89.12 ± 0.49	88.74 ± 0.42
Tumor postoperative risk assessment dataset				
Model variant	Accuracy	Precision	Recall	F1 score
w./o. Low Dimensional Manifold Projection	89.45 ± 0.43	89.01 ± 0.52	88.67 ± 0.55	88.89 ± 0.48
Standard embedding instead of manifold projection	89.82 ± 0.41	89.36 ± 0.50	89.05 ± 0.53	89.21 ± 0.46
w./o. Event driven Segmentation Router	90.12 ± 0.40	89.67 ± 0.49	89.34 ± 0.52	89.56 ± 0.45
Average pooling after segmentation	90.31 ± 0.39	89.86 ± 0.47	89.55 ± 0.50	89.74 ± 0.43
w./o. attention in outcome model	90.44 ± 0.38	89.98 ± 0.46	89.71 ± 0.49	89.88 ± 0.42
w./o. Probabilistic Outcome Modeler	90.56 ± 0.38	90.12 ± 0.47	89.78 ± 0.50	90.01 ± 0.43
Ours	91.23 ± 0.39	90.78 ± 0.48	90.45 ± 0.51	90.67 ± 0.44

**Table 8:** Redesigned ablation study on the deep learning attention mechanisms in healthcare dataset and the nursing quality and tumor risk factors dataset.

Healthcare dataset				
Model variant	Accuracy	Precision	Recall	F1 score
w./o. Low Dimensional Manifold Projection	88.45 ± 0.51	87.89 ± 0.57	87.23 ± 0.60	87.56 ± 0.55
Standard embedding instead of manifold projection	88.76 ± 0.49	88.18 ± 0.55	87.52 ± 0.58	87.84 ± 0.53
w./o. Event driven Segmentation Router	88.78 ± 0.49	88.23 ± 0.55	87.56 ± 0.58	87.89 ± 0.54
Average pooling after segmentation	89.01 ± 0.47	88.46 ± 0.53	87.78 ± 0.56	88.08 ± 0.52
w./o. attention in outcome model	89.08 ± 0.46	88.53 ± 0.52	87.84 ± 0.55	88.17 ± 0.51
w./o. Probabilistic Outcome Modeler	89.12 ± 0.47	88.56 ± 0.53	87.89 0.56	88.22 ± 0.52
Ours	89.78 ± 0.43	89.23 ± 0.49	88.56 ± 0.54	88.89 ± 0.50
Tumor risk factors dataset				
Model variant	Accuracy	Precision	Recall	F1 score
w./o. Low Dimensional Manifold Projection	89.67 ± 0.48	89.12 ± 0.54	88.45 ± 0.59	88.78 ± 0.53
Standard embedding instead of manifold projection	90.02 ± 0.46	89.47 ± 0.52	88.79 ± 0.57	89.12 ± 0.51
w./o. Event driven Segmentation Router	90.12 ± 0.46	89.67 ± 0.52	89.00 ± 0.57	89.34 ± 0.51
Average pooling after segmentation	90.28 ± 0.45	89.81 ± 0.51	89.12 ± 0.55	89.45 ± 0.50
w./o. attention in outcome model	90.37 ± 0.44	89.86 ± 0.50	89.18 ± 0.54	89.51 ± 0.49
w./o. Probabilistic Outcome Modeler	90.45 ± 0.44	89.89 ± 0.50	89.23 ± 0.55	89.56 ± 0.49
Ours	91.12 ± 0.42	90.67 ± 0.48	90.00 ± 0.53	90.34 ± 0.49

### 4.6 Real world clinical cohort validation

To further evaluate the clinical applicability of the proposed framework, an external validation experiment was conducted using a real world clinical cohort collected from a tertiary hospital between January 2021 and December 2023. The cohort included 1,286 patients who underwent tumor related surgical procedures and had complete perioperative nursing records, intraoperative monitoring records, anesthesia records, surgical records, and postoperative follow up information. Patients with missing key perioperative variables, incomplete postoperative follow up, duplicated records, or inconsistent outcome documentation were excluded. The cohort consisted of structured clinical records and perioperative time series data. The structured variables included demographics, comorbidities, tumor characteristics, surgical information, anesthesia type, intraoperative blood loss, transfusion status, laboratory indicators, nursing intervention records, and postoperative observations. The perioperative time series included time stamped vital signs, medication administration, transfusion events, nursing interventions, and postoperative monitoring records.

No image data were used in this cohort validation, and image based augmentation was therefore not applied. The primary outcome was postoperative complication occurrence within 30 days after surgery, including surgical site infection, postoperative bleeding, pulmonary complications, renal complications, unplanned intensive care unit admission, and delayed postoperative recovery. Among the 1,286 patients, 214 patients had at least one documented postoperative complication. The secondary outcome was operating room nursing quality, which was categorized as low, moderate, or high according to institutional nursing quality assessment criteria. In this cohort, 168 cases were classified as low quality, 472 as moderate quality, and 646 as high quality. For external validation, the model was trained on the original training datasets and evaluated on the independent real world clinical cohort. Samples from this cohort were not used for model training. Preprocessing parameters, including imputation values, normalization statistics, and categorical encoding mappings, were estimated from the training set and directly applied to the real world cohort. The same evaluation metrics as the main experiments were used, including accuracy, precision, recall, F1 score, and area under the receiver operating characteristic curve.

As shown in **Table 9**, the external validation cohort represented clinically collected perioperative records rather than a curated benchmark dataset. As shown in **Table 10**, the proposed framework maintained stable predictive performance on the independent clinical cohort. For postoperative complication prediction, the model achieved an accuracy of 86.42%, an F1 score of 84.33%, and an AUC of 0.887. For operating room nursing quality assessment, the model achieved an accuracy of 87.35%, an F1 score of 86.39%, and an AUC of 0.902. The performance on the external cohort was slightly lower than that on the curated datasets, which may be attributed to greater heterogeneity, irregular temporal sampling, and variable documentation patterns in real world clinical records. These results support the potential applicability of the proposed framework to clinically collected perioperative data. However, because the cohort was collected from a single institution, broader prospective multi center validation remains necessary before routine clinical deployment. Therefore, the proposed framework should be regarded as a computational risk assessment approach with promising clinical applicability rather than a fully validated clinical decision making system.

**Table 9:** Characteristics of the real world clinical cohort used for external validation.

Characteristic	Value
Clinical center	A tertiary hospital
Study period	January 2021 to December 2023
Number of patients	1,286
Surgery type	Tumor related surgical procedures
Data modality	Structured clinical records and perioperative time series
Image data	Not included
Main clinical variables	Demographics, comorbidities, tumor characteristics, surgical details, anesthesia information, intraoperative indicators, nursing interventions, laboratory indicators, and postoperative observations
Primary outcome	Postoperative complication occurrence within 30 days after surgery
Number of patients with postoperative complications	214
Secondary outcome	Operating room nursing quality category
Distribution of nursing quality labels	Low quality: 168; moderate quality: 472; high quality: 646
Postoperative complications	Surgical site infection, postoperative bleeding, pulmonary complications, renal complications, unplanned intensive care unit admission, and delayed postoperative recovery
Nursing quality indicators	Nursing documentation completeness, perioperative nursing intervention implementation, intraoperative safety management, infection control compliance, and postoperative handover quality
Validation protocol	External validation without using cohort samples for model training
Preprocessing	Missing value imputation, categorical encoding, temporal alignment, outlier checking, label consistency checking, and normalization
Augmentation	No image based augmentation was applied

**Table 10:** External validation results on the real world clinical cohort.

Task	Accuracy	Precision	Recall	F1 score	AUC
Postoperative complication prediction	86.42 ± 0.48	84.91 ± 0.55	83.76 ± 0.61	84.33 ± 0.57	0.887 ± 0.012
Operating room nursing quality assessment	87.35 ± 0.44	86.72 ± 0.51	86.08 ± 0.53	86.39 ± 0.49	0.902 ± 0.010

## 5 Conclusions and future work

This study proposed the Counterfactual Risk Assessor, a deep learning framework with attention mechanisms for operating room nursing quality assessment and postoperative risk prediction in tumor surgery patients. The framework integrates Low Dimensional Manifold Projection, Event driven Segmentation Router, and Probabilistic Outcome Modeler to learn compact perioperative representations, emphasize clinically relevant event windows, and estimate outcome probabilities with uncertainty information. Counterfactual pacing and uncertainty propagation were further incorporated to examine plausible alternative perioperative scenarios and quantify prediction confidence. Experimental results showed that the proposed framework achieved better predictive performance than clinical, statistical, machine learning, and deep learning baselines across the evaluated datasets. These findings suggest that event aware representation learning and attention based probabilistic modeling may provide useful support for postoperative risk stratification and nursing quality evaluation. However, the results should be interpreted as evidence of potential decision support value rather than direct evidence of improved clinical outcomes. The framework is not intended to replace clinical judgment, and its use in routine care requires further validation.

Several limitations remain. Model performance depends on the quality, completeness, and consistency of perioperative records. Missing or noisy data may affect representation learning and risk estimation, especially in institutions with limited digital documentation. Although attention weights and uncertainty estimates improve transparency to some extent, the interpretability of deep learning based predictions remains limited. The current evaluation is primarily retrospective, and prospective clinical evidence is still needed. Future work will focus on external multicenter validation, prospective testing, and assessment of model performance in real clinical workflows. Additional studies should examine calibration, fairness across patient subgroups, robustness under missing data, and clinical usability by operating room nurses and perioperative clinicians. Further integration with explainable artificial intelligence methods may also help clarify the relationship between perioperative events, nursing interventions, and predicted postoperative risk.

## Data Availability

The original contributions presented in the study are included in the article/supplementary material. Further inquiries can be directed to the corresponding author.
